# Supplementary material for: Comparison of perinatal outcome and mode of birth of twin and singleton pregnancies in migrant and refugee populations on the Thai Myanmar border: A population cohort
Source: PLoS One. 2024 Apr 18;19(4):e0301222. doi: 10.1371/journal.pone.0301222 (PMC11025774; doi:10.1371/journal.pone.0301222)
Supplement: S1 File — (DOCX) [file pone.0301222.s001.docx]

**Supplementary Table 1: Twinning rate in 5-year periods at Shoklo Malaria Research Unit (SMRU) antenatal clinics 1986-2020.**

| Years | Twinning rate ( |
| --- | --- |
| 1986-1990 | 5 per 1000 (12/2,196) [3-10] |
| 1991-1995 | 9 per 1000 (58/6,519) [7-11] |
| 1996-2000 | 10 per 1000 (90/9,405) [8-12] |
| 2001-2005 | 9 per 1000 (115/12,861) [7-11] |
| 2005-2010 | 9 per 1000 (169/19,110) [8-10] |
| 2011-2015 | 8 per 1000 (158/18,902) [7-10] |
| 2016-2020 | 9 per 1000 (116/13,038) [7-11] |

Data are rate per 1000 (n twin pregnancies/N all pregnancies) [95% CI]%

**Supplementary Table 2: Stillbirth proportion (expressed as % (n/n) [95% CI]) according to birth weight discordance in Twin 1 and Twin 2**

|  | Stillbirth twin 1 | Stillbirth twin 2 |
| --- | --- | --- |
| Weight difference Twin 1 and twin 2 less than 40% | 1.3 (7/520) [0.5-2.8] | 3.9 (20/520) [2.2-5.5] |
| Weight Twin 1 > 40% larger than twin 2. | 0 (0/34) [NA] | 14.7 (5/34) [4.4-28.1] |
| Weight Twin 2 > 40% larger than twin 1. | 11.1 (2/18) [1.4-34.7] | 5.6 (1/18) [0.1-27.3] |

Data % (n stillbirth/ N twin pregnancy) [95% CI]

**Supplementary Table 3: Stillbirth rate by place of birth based on place of birth twin 1**

|  | Twin pregnancy | | Singleton pregnancy |
| --- | --- | --- | --- |
|  | Twin 1 | Twin 2 |  |
| SMRU | 28 per 1000 (9/327) | 67 per 1000 (22/327) | 9 per 1000 (304/34,340) |
| Home/Other | 61 per 1000 (5/82) | 98 per 1000 (8/82) | 7 per 1000 (97/13,574) |
| Hospital | 6 per 1000 (1/177) | 45 per 1000 (8/178) | 33 per 1000 (158/4,749) |

Data stillbirth per 1000 births (n/N)

Missing data for place of birth: n= Twin 14, n= Singleton 6,342

**Supplementary Table 4: Early Neonatal Death per category of estimated gestational age at birth**

| Estimated Gestational Age | Twin 1 | Twin 2 | Singleton |
| --- | --- | --- | --- |
| 28 - <32 weeks | 40.5 (17/42) | 39.0 (16/41) | 29.3 (135/461) |
| 32- <37 weeks | 4.3 (9/208) | 5.0 (10/199) | 3.6 (168/4,619) |
| ≥37 weeks | 0.6 (2/330) | 0.6 (2/317) | 0.5 (271/53,021) |

Data are % (n/N)

**Supplementary Table 5: Early Neonatal Death by mode of delivery of twin 1**

|  |  | Twin 1 | Twin 2 |
| --- | --- | --- | --- |
| First twin Vaginal birth | Any EGA | 5.3 (24/452) [3.4-7.9] | 6.0 (26/433) [3.9-8.8] |
|  | ≥28 - <32 weeks | 45.9 (17/37) [26.8-73.6] | 44.4 (16/36) [25.4-72.2] |
|  | ≥32- <37 weeks | 3.1 (5/161) [1.0-7.2] | 5.0 (8/155) [2.2-10.1] |
|  | ≥37 weeks | 0.8 (2/254) [0.1-2.8] | 0.8 (2/242) [0.1-3.0] |
| First twin caesarean | Any EGA | 3.1 (4/128) [0.8-8.0] | 1.6 (2/124) [0.2-5.8] |
|  | ≥28 - <32 weeks | 0 (0/5) [NA] | 0 (0/5) [NA] |
|  | ≥32- <37 weeks | 8.5 (4/47) [2.3-21.8] | 4.5 (2/44) [0.5-16.4] |
|  | ≥37 weeks | 0 (0/76) [NA] | 0 (0/75) [NA] |

Data are % (n/N) [95 CI]

**Supplementary Table 6: Proportion of maternal death over three decades for twin and singleton pregnancies**

|  | % Maternal Death (Maternal Death/total births) | |
| --- | --- | --- |
|  | Twin | Singleton |
| 1991-2000 | 0 (0/137) | 0.1 (16/13,584) |
| 2001-2010 | 0 (0/232) | 0.1 (21/21,982) |
| 2011-2020 | 1 (3/219) | 0.1 (20/21,954) |

Data are % (n/n) [95 CI]
