# Supplementary material for: Comparison of perinatal outcome and mode of birth of twin and singleton pregnancies in migrant and refugee populations on the Thai Myanmar border: A population cohort
Source: PLoS One. 2024 Apr 18;19(4):e0301222. doi: 10.1371/journal.pone.0301222 (PMC11025774; doi:10.1371/journal.pone.0301222)
Supplement: Attachment — Submitted filename: Perinatal outcome of twin pregnancy.pdf [file pone.0301222.s002.pdf]

12 October 2023

Dear Sir,

I have reviewed the manuscript entitle of “Perinatal outcome of twin pregnancy and mode of birth in marginalized migrant and refugee populations on the Thai Myanmar border: a population cohort”.

I think, this work is a big retrospective data and it can be represent the border health where access to hospital care is limited. The result is interesting which can be used to monitor and manage the pregnant women in the remote area.

I would like to make a decision for accept this work. I am not sure that I misunderstand for the number of cases in table 3, please check the number of twin pregnancy (n=595) but in the detail: birthed vaginally is 597.   Line 231, 2946 grams should be 2,946 grams.

Sincerely,
